# Multi-Criteria Decision Analysis for Benefit-Risk Analysis by National Regulatory Authorities

**DOI:** 10.3389/fmed.2021.820335

**Published:** 2022-01-12

**Authors:** Orin Chisholm, Patrick Sharry, Lawrence Phillips

**Affiliations:** ^1^PharmMed, Sydney, NSW, Australia; ^2^Edson College of Nursing and Health Innovation, Arizona State University, Tempe, AZ, United States; ^3^People and Decisions, Sydney, NSW, Australia; ^4^The University of New South Wales (UNSW) Sydney, Sydney, NSW, Australia; ^5^Decision Science, London School of Economics and Political Science, London, United Kingdom

**Keywords:** benefit-risk, regulatory science, decision analysis, regulatory affairs, drug approval process

## Abstract

The approval process for pharmaceuticals has always included a consideration of the trade-offs between benefits and risks. Until recently, these trade-offs have been made in panel discussions without using a decision model to explicitly consider what these trade-offs might be. Recently, the EMA and the FDA have embraced Multi-Criteria Decision Analysis (MCDA) as a methodology for making approval decisions. MCDA offers an approach for improving the quality of these decisions and, in particular, by using quantitative and qualitative data in a structured decision model to make trade-offs in a logical, transparent and auditable way. This paper will review the recent use of MCDA by the FDA and EMA and recommend its wider adoption by other National Regulatory Authorities (NRAs) and the pharmaceutical industry.

## Introduction

Many national regulatory authorities (NRAs) make decisions regarding the approval of new medicinal products using a risk-based approach. However, they differ in the methodologies used to weigh up the benefits, or desirable outcomes, of a medicine compared with the risks, or undesired effects of the medicine (1). Indeed, many NRAs still rely on mostly qualitative approaches to benefit-risk decision-making. With the recent trends towards greater transparency and inclusion of the patient voice in regulatory decision-making, NRAs are moving towards more structured, quantitative methods to support their benefit-risk decisions. While each NRA still maintains the right to make these decisions based on the individual circumstances that apply within their country, more structured approaches will help contribute towards greater harmonisation and enable product developers greater certainty in making their own internal decisions during the development of new medicines.

## Benefit-Risk Analysis in Regulatory Decision-Making

The science of benefit-risk analysis is rapidly evolving and there is no one consensus method in use by various NRAs (2). Benefit-risk decisions made by NRAs take into account the efficacy and safety evidence provided by the sponsor as well as the nature of the disease being treated, the availability of other treatments for that disease and the ability to ensure the benefits outweigh the risks by utilising appropriate risk management tools (3–6). The benefit-risk determination requires a thorough investigation of the evidence, recognition of evidence gaps and careful consideration of a wide range of factors. Many NRAs have developed a qualitative framework for assessing the benefit-risk profile of medicines. In some cases, the benefit-risk profile is relatively straightforward to determine but in others it can be difficult to make an appropriate judgement among the benefits and risks for the population. In such cases, NRAs may resort to more quantitative methodologies to determine the benefit vs. risk balance.

The use of structured benefit-risk evaluations by NRAs has increased in line with the maturing of the benefit-risk science (7–9). In 2012, the International Council for Harmonisation of Technical Requirements for Pharmaceuticals for Human use (ICH) updated the Periodic Safety Update Report (PSUR) to the Periodic Benefit-Risk Evaluation Report (PBRER), which placed greater emphasis on the determination of the benefit-risk assessment for medicines, rather than just acting as a safety update report (10). As the new guideline (11) has been gradually adopted by NRAs, an increase in reliance on formal benefit-risk assessment procedures has been observed (12). The PBRER explicitly notes that formal quantitative benefit-risk analysis may be considered in the benefit-risk evaluation of a medicine and that the methodology used should be included in the PBRER report.

### Qualitative and Quantitative Assessment Methods

The Pharmaco-epidemiological Research on Outcomes of Therapeutics by a European Consortium (PROTECT) project was established in 2009 under the Innovative Medicines Initiative (IMI) and the project ran until 2015 (13). One of the outcomes of the project was to review various benefit-risk frameworks (2, 14). These were classified as either descriptive or quantitative. Descriptive frameworks included PrOACT-URL (14), Benefit Risk Action Team (BRAT) (15), Ashby and Smith framework (16), Consortium On Benefit and Risk Assessment (COBRA) framework (17), the FDA's Benefit-Risk framework (18), and a Universal Methodology for Benefit-Risk Assessment (UMBRA) (19). Quantitative frameworks included Multicriteria Decision analysis (MCDA) (20), Stochastic Multi-attribute Acceptability Analysis (SMAA) (21), decision tree model (22), and Markov decision process (23). The outcome of the project was a recommendation for further testing and consideration of the PrOACT-URL, BRAT, MCDA and SMAA frameworks in benefit-risk determinations.

Recently, Kurzinger et al. (7) reviewed the published literature on the use of structured benefit-risk assessment methods in drug development. They conclude that regulators and industry are increasingly relying on descriptive frameworks supplemented by quantitative methods. However, there is still confusion on how and when to use these frameworks and how to integrate patient perspectives in the benefit-risk process.

### Multi-Criteria Decision Analysis (MCDA)

Participants in the IMI-PROTECT project were free to use, independently, any of the frameworks in testing the different approaches, but it is notable that all six of the drugs they modelled applied MCDA. This is the approach we will elaborate here. Multi-criteria analysis (MCA) is a way of helping people manage complex decision-making processes. MCA techniques can be used to identify a preferred option, rank those options or distinguish an acceptable from an unacceptable option (20). Multi-criteria decision analysis (MCDA) is one form of MCA. MCDA aims to provide an order to various options, from most preferred to least preferred. It acts as an aid to thinking and decision-making and enables greater coherence in the decision-making process. The core technical elements of an MCDA model are the alternatives (e.g., a drug at varying doses vs. a placebo), the criteria (the individual attributes that will be used to determine if one alternative is better than another) and weights (which reflect the relative importance of the attributes once they have been scored). A simple algorithm that is soundly based on decision theory (24) is then used to combine these scores and produce an ordered list. MCDA also addresses the social elements of the decision process. By making the scoring of each alternative against the criteria an explicit process, it encourages discussion amongst decision makers in a productive way. This is particularly important in situations where data are limited, or the implications of the data are unclear. It also allows for the inclusion of the views of diverse stakeholder groups, such as patient groups (7). The same process applies to the determination of weights. Furthermore, if there are differences of opinion these can be addressed through sensitivity analysis which determines whether those differences are material and if so helps to resolve those differences. The process can be summarised as shown in Table 1.

**Table 1:** High level MCDA process [adapted from (20)].

Define problem	
Agree alternatives (options)	Agree criteria
Score alternatives against criteria	
Weight criteria	
Combine scores and weights using a simple algorithm to produce an overall score for each alternative	
Conduct sensitivity analysis	
Produce final recommendation	

## Case Studies of Recent Decisions

### EMA

The European Medicines Agency (EMA) undertook a benefit-risk project in 2009–2011 with the aim to improve the way committees' assessment reports present benefit-risk assessments. This project resulted in the development of internal tools for determining and presenting benefit-risk and the inclusion of a benefit-risk effects table in the assessment report documents. The EMA benefit-risk project determined that decision analysis provided a theoretically sound basis for quantifying favourable and unfavourable effects, as well as clinical relevance and its associated uncertainties. A review of different methodologies for determining benefit-risk concluded that MCDA was a “logical, coherent model for decisions with multiple objectives” and could handle uncertainties (25). From this the EMA selected the PrOACT-URL descriptive framework. An overview of this model is depicted in Table 2.

**Table 2:** PrOACT-URL framework [adapted from (26)].

**Step**	**Details**
Problem	Determine the nature of the problem and its context
Objectives	Identify objectives that indicate the overall purposes to be achieved
Alternatives	Identify the options to be evaluated against the criteria
Consequences	Describe how the alternatives perform on the criteria, ie, the magnitude of the favourable and unfavourable effects
Trade-offs	Assess the balance between the favourable and unfavourable effects
Uncertainty	Consider how the balance changes when taking into consideration the uncertainty associated with the consequences. This may be facilitated using a secondary effects table.
Risk Attitude	Judge the relative importance of the Agency's risk attitude for the medicine under consideration
Linked decisions	Consider the consistency of the decision with past decisions

The EMA has used this model in their decision-making processes since then. They supplement it with quantitative methods such as MCDA, particularly when the benefit-risk decision is complex and there are many factors to take into consideration in coming to their conclusion (27, 28). One example where they have used PrOACT-URL supplemented with MCDA was when they were reviewing the extension of indications for quadrivalent human papillomavirus recombinant vaccine (Gardasil) to include “prevention of premalignant anal lesions and anal cancer” (29). The sponsor included a PrOACT-URL/MCDA analysis to support their proposal that the benefits of vaccination in the general population outweighed the potential risks associated with the vaccine in males. This approach was used to overcome limitations in the “number needed to vaccinate” vs. the “number needed to harm” metrics commonly used to evaluate vaccines. The MCDA modelling compared the vaccine to no vaccination. External experts were consulted in building the model. Benefit and risk data were transformed linearly into 0–100 preference values and criteria were weighted to ensure equal units on all scales so that sums of the weighted scores could compare the vaccine to no vaccine. The model suggested a superior benefit-risk score of 66 for the vaccine compared with 46 for no vaccination. Various sensitivity analyses were performed on the model but they resulted in little change to the overall benefit-risk scores (29).

Marcelon et al. (30) modelled a quantitative benefit-risk assessment of the same quadrivalent human papillomavirus recombinant vaccine in males for the additional indication of preventing anal cancer. They established a decision context to define the objective of the assessment then identified the key benefits and risks in a value tree and developed an effects table, weighed the various values and used that to determine the benefit-risk balance. Their analysis showed a positive benefit-risk balance with prevention of anal cancer and genital warts being the most beneficial effects. Increasing serious adverse effects to hypersensitivity reactions did not appreciably alter the benefit-risk balance for the vaccine. The benefits of the MCDA methodology were apparent with a clear ability to make the way benefits and risks are assessed much more transparent (30).

### FDA

The FDA uses a structured benefit-risk framework based on an analysis of the evidence and uncertainties for four dimensions: analysis of the condition, current treatment options, benefit, risk and risk management (31–33). The framework will be supported by a guidance document on benefit-risk assessment for new drug and biological products, which is currently undergoing consultation (18, 34). The focus of this framework is on qualitative analysis, but more recently the FDA has applied structured quantitative approaches, such as MCDA, to challenging decisions. Indeed, the FDA has established a Decision Support and Analysis Team within the Office of Program and Strategic Analysis to support benefit-risk analysis within the organisation.

In a recent decision on ticagrelor, for the first time the FDA applied MCDA to develop a more robust decision model (34–36). The process followed the steps set out in Table 1 above. Ticagrelor had originally been approved by the FDA in 2011 for secondary prevention of cardiovascular events. The 2019 THEMIS (The Effect of Ticagrelor on Health Outcomes in Diabetes Mellitus Patients Intervention Study) project provided additional data that led to the approval of ticagrelor for reducing the risk of first myocardial infarction in patients with coronary artery disease. However, the benefit-risk balance was not clear. The FDA applied MCDA to assist with clarifying the sensitivities involved in the decision. The analysis focused on the performance of the drug and the placebo on six criteria (cardiovascular death, myocardial infarction, ischemic stroke, fatal bleed, intracranial haemorrhage and other major bleeds). The effect of ticagrelor and the placebo were scored on each of these criteria, using data from the THEMIS project. A group of experts worked together on the weighting step in the MCDA process to develop explicit quantitative trade-offs between these criteria. Combining the weights and the criteria scores in the MCDA algorithm produced an overall value for the alternatives. Importantly, because all steps involved explicit quantitative judgements, a sensitivity analysis could easily be conducted to explore contentious points in greater detail. The overall conclusion of the MCDA analysis showed that ticagrelor could be used for primary prevention in patients with high risk of cardiovascular events, and it subsequently was approved.

Lackey (35) contains important insights into the value of the MCDA approach taken by the FDA: data could be used to score performance of the alternatives against the criteria; the process allowed a diverse group of experts to work together to develop weights that represented the trade-offs between the criteria; assumptions could be identified and tested; sensitivity analysis provided deeper insights into the issues, especially the trade-offs between criteria; and the process provided a level of transparency not usually available when trade-offs are implicit.

## MCDA in Drug Development

As a quantitative and structured form of decision-making, MCDA has significant applicability for including the patient voice in regulatory decisions. There has been a rise in the call for great inclusion and visibility of the patient perspective in regulatory processes due to their unique perspectives on living with a disease (37–39). The ICH has recently released a reflection paper on the need for NRAs and drug developers to incorporate the patient perspective during early-stage drug development and throughout the lifecycle of the medicine (40). This will also feed into the revision of the Good Clinical Practise (GCP) guideline as part of their GCP renovation project (41). Of importance to the benefit-risk decision is the incorporation of the patient's perspectives on the benefits and the adverse effects that matter most to them.

The EMA has a well-established framework for the incorporation of the patient perspective into the regulatory decision-making process and provide an annual report on stakeholder engagement (42). They have recently hosted a symposium on new approaches in patient-focused cancer drug development, with a focus on generation and incorporation of real-world evidence into the regulatory decision-making process (43).

The FDA has focused on patient focused drug development in recent years following the twenty-first Century Cures Act and the FDA Reauthorization Act, both of which required the FDA to do so. Specifically, Sections 3001-3004 of the twenty-first Century Cures Act requires the FDA to include a statement regarding patient experience data used at the time of registration, produce guidance documents on how to collect patient experience data to be used for benefit-risk evaluation of therapeutic products and for the FDA to make reports on their use of patient experience (44). As a result, the FDA has established a number of guidances and information on their website (45, 46).

Additionally, MCDA has practical use in conjunction with various machine learning algorithms for big data analysis in early stage drug development (47–49); as a tool for making critical decisions during drug development (50–52); in health technology assessment decisions (53, 54) or to aid patient decisions regarding their choice of treatment (55). Structured benefit-risk frameworks are beginning to be utilised more often by industry (56, 57), although a lack of a globally-harmonised framework has limited diffusion of structured benefit-risk decision by industry (58).

## Recommendations

We propose the use of an MCDA-based framework in benefit-risk decision making by national regulatory authorities in a move towards greater harmonisation of the benefit-risk assessment across jurisdictions. This will provide greater certainty for sponsors of medicines and aid them in developing their registration dossiers for new products.

We recommend that sponsors and others involved in the development of new medicines should utilise such structured decision-making when developing new medicines as this will facilitate the streamlining of innovation in medicines development. Greater structure around critical “go/no-go” decision points during product development will help organisations make rational decisions about whether they should proceed, abandon or re-focus their development programs.

We recommend that MCDA be utilised to provide a quantitative way of incorporating the patient voice into drug development and regulatory decisions.

## Discussion

The examples above demonstrate the potential for MCDA as an approach to benefit-risk decision making by NRAs and by others involved in pharmaceutical development. The main advantages of MCDA are:

The MCDA process is transparent. Alternatives and criteria are clearly specified. Judgements at all stages (scoring of alternatives against criteria, weighting, sensitivity analysis) are quantitative and explicit. Sensitivity analyses explore the extent to which any individual disagreements affect the final results, which enables the group to agree about the output while preserving individual differences in the inputs. All inputs to an MCDA are the products of the decision conference group and not attributable to any one individual participant in the decision conference.

The MCDA process is auditable and updatable. Any interested stakeholder can examine the process at any point and review each step in the process. Specifically, any trade-offs (e.g., about the relative importance of criteria) are captured quantitatively in the model and can be analysed. Also, as new data becomes available, it can easily be incorporated into the model.

The MCDA process can include a broad range of inputs (e.g., clinical data, proxy data, patient experience, uncertainty). This is particularly valuable for those wishing to include patient experience data explicitly into the decision process. It is very useful in situations where experts need to work with imperfect or incomplete data sets. In these situations, subjective judgements can be made explicitly rather than implicitly.

The MCDA process leverages a contemporary understanding of human cognitive processes. It allows experts to do what they do best (make explicit judgements of effects against clear criteria and make trade-offs about the relative importance of criteria) and uses a simple algorithm to combine these judgements in a transparent way.

The MCDA process assists groups of experts to arrive at conclusions by forcing explicit judgements so that points of agreement and differences in opinion can be easily seen and discussed. Sensitivity analysis allows more detailed analysis of points of difference to surface assumptions that underpin judgements and to make the final decision process more robust.

MCDA has been used in the pharmaceutical domain for several decades, but only recently has it been adopted by NRAs for benefit-risk decisions. Application of this approach has also increased in other parts of the pharmaceutical and medical device development process. As more examples emerge, there will be the opportunity to share learning and to develop best practise for NRA benefit-risk decisions and for other uses. Because of the transparency that is a hallmark of the MCDA process, there is the chance to harmonise approaches across geographies which would assist with the efficiency and robustness of decision making for all involved in drug development.

## Data Availability Statement

The original contributions presented in the study are included in the article/supplementary material, further inquiries can be directed to the corresponding author.

## Author Contributions

OC and PS conceived the article. All authors contributed to the writing of the article and approved the version submitted.

## Conflict of Interest

The authors declare that the research was conducted in the absence of any commercial or financial relationships that could be construed as a potential conflict of interest.

## Publisher's Note

All claims expressed in this article are solely those of the authors and do not necessarily represent those of their affiliated organizations, or those of the publisher, the editors and the reviewers. Any product that may be evaluated in this article, or claim that may be made by its manufacturer, is not guaranteed or endorsed by the publisher.
